# Positive end-expiratory pressure titration in COVID-19 acute respiratory failure: electrical impedance tomography vs. PEEP/FiO_2_ tables

**DOI:** 10.1186/s13054-020-03242-5

**Published:** 2020-09-01

**Authors:** Nicolò Sella, Francesco Zarantonello, Giulio Andreatta, Veronica Gagliardi, Annalisa Boscolo, Paolo Navalesi

**Affiliations:** 1 Department of Medicine - DIMEDD, University of Padua, via V. Gallucci 13, 35125 Padua, Italy; 2 Anaesthesia and Intensive Care Unit, Padua University Hospital, via V. Gallucci 13, 35125 Padua, Italy

**Keywords:** COVID-19, Acute respiratory failure, Mechanical ventilation, Positive end-expiratory pressure, Electrical impedance tomography

To the Editor,

Hypoxemic acute respiratory failure (hARF) secondary to COVID-19 presents with heterogeneous features depending on several determinants, such as the extent of intravascular microthrombosis, superinfections, and other complications [1, 2]. The easiest approach for setting positive end-expiratory pressure (PEEP) and inspiratory oxygen fraction (FiO_2_) is using PEEP/FiO_2_ tables [3, 4]. However, because the magnitude of lung recruitability is variable, personalizing PEEP would be desirable [1]. Electrical impedance tomography (EIT) offers this opportunity by bedside estimating both alveolar collapse and lung overdistension throughout a decremental PEEP trial [5].

This investigation (Ethics Committee approval: Ref:4853/AO/20-AOP2012) aims to assess the agreement between EIT-based PEEP values and those recommended by the higher and lower PEEP/FiO_2_ tables [6] in a series of consecutive intubated COVID-19 hARF patients, admitted to intensive care unit at our institution. Written informed consent was obtained from all patients.

We performed 15 decremental PEEP trials through a dedicated device (Pulmovista500, Drӓger-Medical, Germany) and subsequently analyzed pulmonary perfusion distribution [5]. Five patients were evaluated in a prone position. EIT optimal PEEP (PEEP_EIT_) was defined as the best compromise between lung collapse and overdistension [5]. All patients were deeply sedated without spontaneous breathing efforts and ventilated in volume control mode with lung-protective settings [3]. PEEP_EIT_ was compared with PEEP from higher and lower PEEP/FiO_2_ tables [6]. Data, expressed as median and interquartile ranges or 95% confidence interval (CI), were analyzed with the Mann–Whitney test for comparisons and Spearman rank test for correlations, considering *p* values < 0.05 significant. The Bland–Alman analysis was also performed.

Patients had received invasive ventilation for 12.0 (10.0–14.5) days. Patients’ age was 63 (56–78) years, while body mass index (BMI) was 26.2 (25.4–30.9) kg/m^2^. Pulmonary shunt and dead space, as assessed by EIT [5], were 4% (2–6%) and 27% (23–36%), respectively. d-dimer was increased [759 (591–1208) mcg/L], while procalcitonin blood concentration was nearly normal [0.53 (0.34–0.70) mcg/L]. PEEP_EIT_ was 12 (10–14) cmH_2_O and was significantly different from PEEP values of both higher [17 (16–20) cmH_2_O, *p* < 0.001] and lower [9 (8–10) cmH_2_O, *p* = 0.049] PEEP/FiO_2_ tables. The Bland–Altman analysis showed that PEEP_EIT_ was 6.2 [CI 3.9–8.4] cmH_2_O smaller and 2.0 [CI 0.1–4.0] cmH_2_O greater than PEEP levels recommended, respectively, by the higher and lower PEEP/FiO_2_ tables (Fig. 1). No correlation was found between PEEP_EIT_ and FiO_2_ (*p* = 0.789) (Fig. 2). The loss of lung compliance secondary to lung collapse observed with PEEP values from the lower PEEP/FiO_2_ table [7.0% (3.2–8.7%)] was not significantly greater, compared to that obtained with PEEP_EIT_ [3.0% (2.0–4.7%)] (*p* = 0.077). Conversely, the loss of lung compliance consequent to lung overdistension was significantly greater with PEEP values from the higher PEEP/FiO_2_ table [15.5% (11.0–21.5%)] than with PEEP_EIT_ [4.0% (3.0–4.7%)] (*p* < 0.001).

**Fig. 1:**
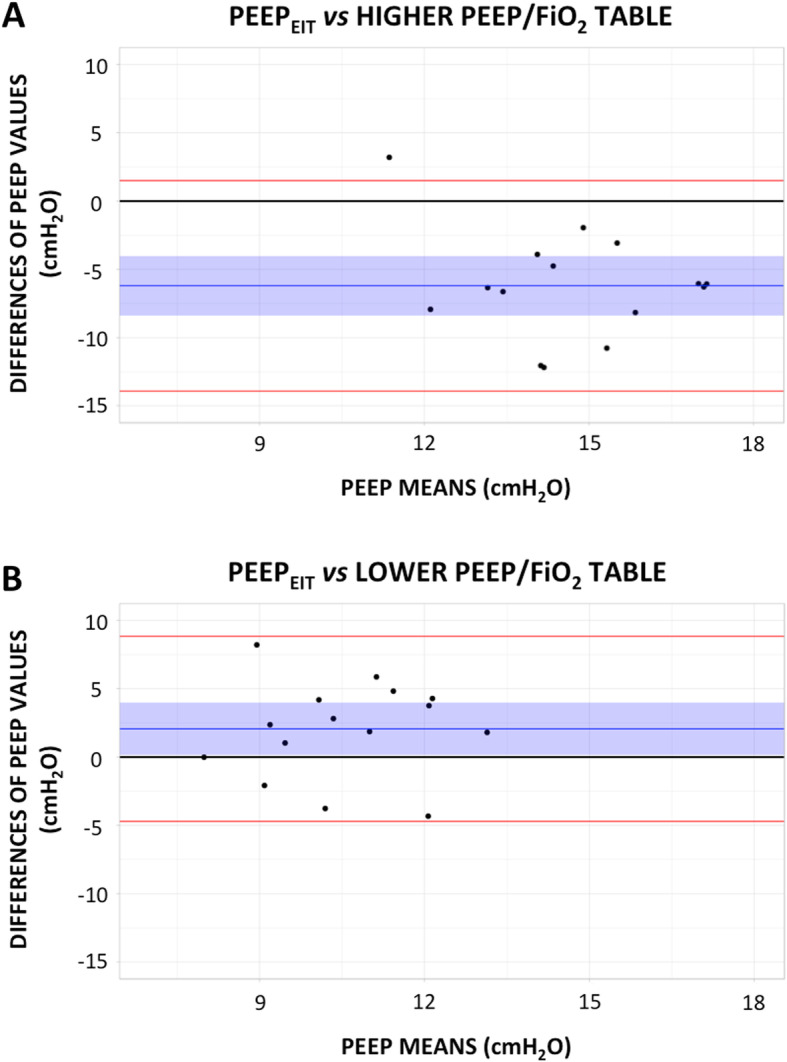
Bland–Altman plot, evaluating the agreement between PEEP_EIT_ and the PEEP values proposed by the higher (**a**) and lower (**b**) PEEP/FiO_2_ tables from the ALVEOLI trial [6]. *X*-axis: average of paired measurements. *Y*-axis: difference between paired measurements. The blue line and blue shaded area: bias and 95% confidence interval of the bias between PEEP_EIT_ and the PEEP values suggested by PEEP/FiO_2_ tables. Red lines: upper and lower limits of agreement between methods

**Fig. 2:**
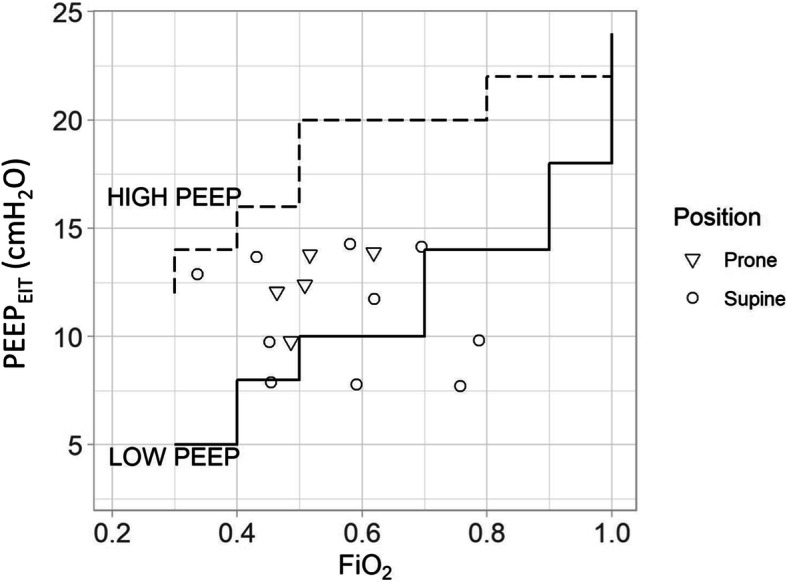
Spearman correlation between PEEP_EIT_ and FiO_2_ (*R* = 0.075, *p* = 0.789). Continuous line: lower PEEP/FiO_2_ table. Dashed line: higher PEEP/FiO_2_ table

In contrast to our results, a recent study, utilizing the same EIT device in intubated COVID-19 hARF patients, reported much higher values of PEEP_EIT_ [21 (16–22) cmH_2_O], closer to those indicated by the higher PEEP/FiO_2_ table, though without significant correlation [4]. These differences are partly explained by the different criteria for PEEP_EIT_ selection, which in that study was set above the value indicated by the built-in algorithm corresponding to the least lung collapse and overdistension [4]. Also, compared to our study, they enrolled more obese patients, as indicated by the higher BMI [30.0 (27.0–34.0) kg/m^2^] [4]. Not reported in that study [4], our patients showed increased d-dimer and high fraction of pulmonary dead space, while shunt fraction and procalcitonin were nearly normal, suggesting predominant lung vascular disruption.

In conclusion, we confirm the rationale for individualized PEEP setting in COVID-19 patients intubated for hARF. Whether EIT is the best technique for this purpose and the overall influence of personalizing PEEP on clinical outcome remain to be determined.

## Data Availability

The data that support the findings of this study are available from the corresponding author, PN, upon request.
